# eggNOG 4.5: a hierarchical orthology framework with improved functional annotations for eukaryotic, prokaryotic and viral sequences

**DOI:** 10.1093/nar/gkv1248

**Published:** 2015-11-17

**Authors:** Jaime Huerta-Cepas, Damian Szklarczyk, Kristoffer Forslund, Helen Cook, Davide Heller, Mathias C. Walter, Thomas Rattei, Daniel R. Mende, Shinichi Sunagawa, Michael Kuhn, Lars Juhl Jensen, Christian von Mering, Peer Bork

**Affiliations:** 1 Structural and Computational Biology Unit, European Molecular Biology Laboratory, Heidelberg, Germany; 2 Institute of Molecular Life Sciences, University of Zurich, Zurich 8057, Switzerland; 3 Bioinformatics/Systems Biology Group, Swiss Institute of Bioinformatics (SIB), Zurich 8057, Switzerland; 4 The Novo Nordisk Foundation Center for Protein Research, Faculty of Health and Medical Sciences, University of Copenhagen, Copenhagen N 2200, Denmark; 5 Institute of Bioinformatics and Systems Biology, Helmholtz Zentrum München, German Research Center for Environmental Health (GmbH), Neuherberg 85764, Germany; 6 CUBE—Division of Computational Systems Biology, Department of Microbiology and Ecosystem Science, University of Vienna, Vienna 1090, Austria; 7 Daniel K. Inouye Center for Microbial Oceanography: Research and Education, University of Hawaii, Honolulu, HI 96822, USA; 8 Max Planck Institute of Molecular Cell Biology and Genetics, Dresden 01307, Germany; 9 Germany Molecular Medicine Partnership Unit (MMPU), University Hospital Heidelberg and European Molecular Biology Laboratory, Heidelberg 69117, Germany; 10 Max Delbrück Centre for Molecular Medicine, Berlin 13125, Germany

## Abstract

eggNOG is a public resource that provides Orthologous Groups (OGs) of proteins at different taxonomic levels, each with integrated and summarized functional annotations. Developments since the latest public release include changes to the algorithm for creating OGs across taxonomic levels, making nested groups hierarchically consistent. This allows for a better propagation of functional terms across nested OGs and led to the novel annotation of 95 890 previously uncharacterized OGs, increasing overall annotation coverage from 67% to 72%. The functional annotations of OGs have been expanded to also provide Gene Ontology terms, KEGG pathways and SMART/Pfam domains for each group. Moreover, eggNOG now provides pairwise orthology relationships within OGs based on analysis of phylogenetic trees. We have also incorporated a framework for quickly mapping novel sequences to OGs based on precomputed HMM profiles. Finally, eggNOG version 4.5 incorporates a novel data set spanning 2605 viral OGs, covering 5228 proteins from 352 viral proteomes. All data are accessible for bulk downloading, as a web-service, and through a completely redesigned web interface. The new access points provide faster searches and a number of new browsing and visualization capabilities, facilitating the needs of both experts and less experienced users. eggNOG v4.5 is available at http://eggnog.embl.de.

## INTRODUCTION

Orthology and paralogy are central concepts in evolutionary biology. They allow distinguishing between molecular sequences that, despite sharing a common ancestry, evolved by different mechanisms: orthologs are the result of speciation events, whereas paralogs originate from gene duplications. This distinction is widely used in molecular biology, since the evolutionary forces shaping the respective classes of sequences are profoundly different and impact the analysis of functional divergence (1). It is generally assumed that orthologous genes are more likely to conserve their function than paralogs, which, in contrast to orthologs, are partially released from selective pressures after duplication. This idea is commonly referred as the Ortholog Conjecture and, although recently questioned (2,3), it is still considered generally valid and represents the basis of most functional annotation methods (4). Consequently, precise orthology assignments are crucial in many fields such as phylogenetics, pharmacology and comparative genomics. However, due to the intricate evolution of most gene families, which often involves multiple nested duplications, genomic rearrangements and horizontal gene transfers, orthology prediction remains as a highly challenging task (4,5), both analytically and computationally.

Therefore multiple orthology resources have been developed that provide precomputed predictions, each based on a different methodology and organism range, and all having different strengths and weaknesses (6,7). The inference approaches fall into two main categories, namely graph-based (8–15) and tree-based (16–19) methods. Graph-based algorithms allow analysis of more species at once and produce groups of orthologous sequences with the common ancestor defined by the set of species considered at the taxonomic level. Tree-based approaches, by contrast, provide finer resolution (i.e. using tree topology to identify specific speciation and duplication events), but they require heavier computations and are more sensitive to methodological artifacts (20).

We maintain a database of Orthologous Groups (OGs) and functional annotations called eggNOG (evolutionary genealogy of genes: Non-supervised Orthologous Groups) (21). eggNOG uses a graph-based unsupervised clustering algorithm extending the COG methodology (22) to produce genome wide orthology inferences, which are further adjusted to provide lineage specific resolution. The database currently covers 2031 eukaryotic and prokaryotic organisms, as well as precomputed mappings for 1655 additional prokaryotes (12). The present manuscript describes the most recent release of eggNOG (*v4.5*, 2015), featuring a number of improvements over its previous release. The most notable ones include (i) modifications to the clustering algorithm in order to make OGs hierarchically consistent across taxonomic levels, (ii) improved annotation of OGs, (iii) the availability of HMM-based tools for fast protein sequence assignment to OGs, (iv) the addition of viral OGs, (v) the availability of fine-grained orthology inferences derived from phylogenetic analysis, (vi) a completely re-designed web interface and (vii) programmatic access through a RESTful Application Programming Interface (API). eggNOG v4.5 is available at http://eggnog.embl.de.

## OVERVIEW OF THE COMPUTATIONAL PIPELINE

**Figure 1.**
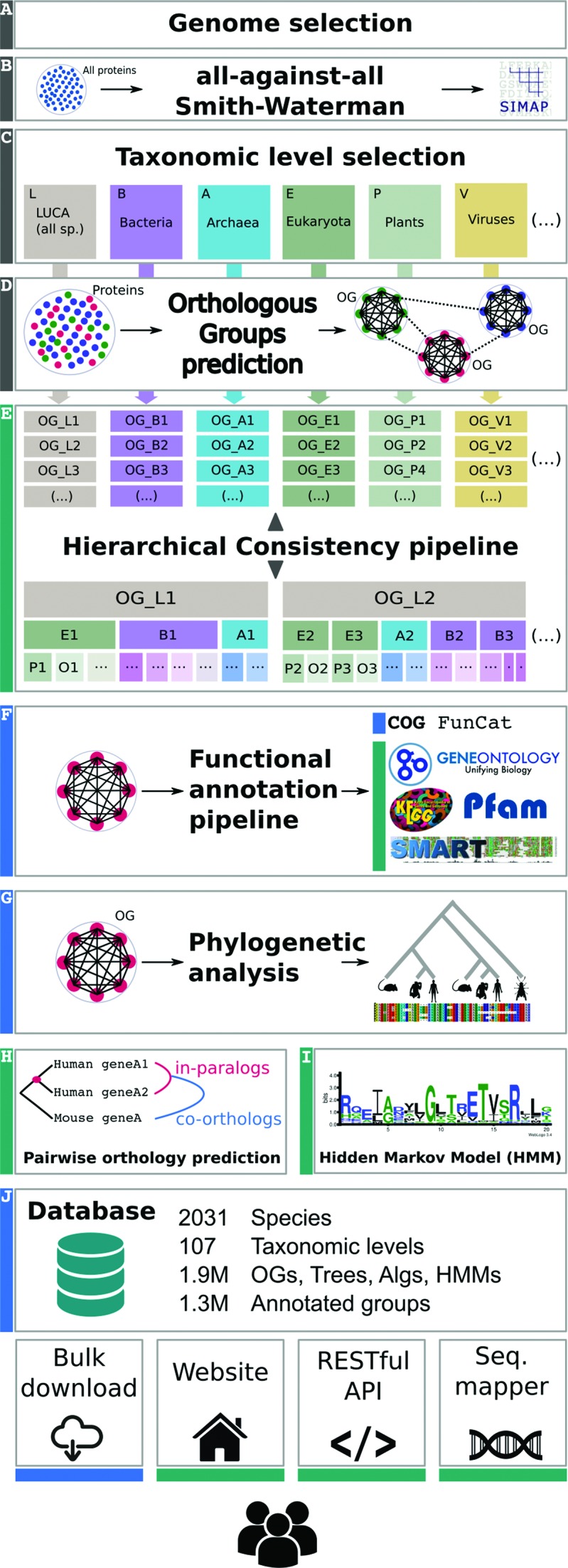
Schematic representation of the eggNOG pipeline: Boxes labelled in green indicate new data and/or methods added in this version. Blue labels represent updated methodology and/or data with respect to previous versions. Grey boxes indicate unchanged steps in version 4.5.

Apart from the central graph-based clustering algorithm, the eggNOG production pipeline involves a number of quality controls as well as pre- and post-processing steps, which have evolved over the last 8 years since its first publication (21). Given the amount of change accumulated at present and previous versions, we describe here the current status of the complete pipeline (Figure 1), highlighting the most recent updates and additions since release 4.0 (12).

### Data set preparation and pairwise sequence comparison

The workflow starts by collecting genomes from public databases (19,23–26). Genomes and proteomes are downloaded, parsed and subjected to quality controls that prevent the inclusion of partial or draft genomes (Figure 1A). This step is coordinated with the STRING (27) and STITCH (28) databases so that the underlying set of protein sequences and names is shared among all three resources. The new viral proteins included in eggNOG v4.5 were retrieved by selecting all reference viral proteomes in Uniprot via XML download on 31 August 2015. These proteomes were filtered by a series of quality controls, which removed 50 proteomes. Eight additional proteomes were included following manual review. Viral proteins are often translated as a single polyprotein, which is cleaved to form functional proteins. Prior to inclusion in eggNOG, such polyproteins were cleaved *in silico* following the ‘chain’ annotations present in Uniprot entries, and only the smallest units were retained, so that the protein sequences are non-redundant.

### Pairwise sequence comparison

Protein sequences from the selected organisms and viruses are extracted and used to compute an *all-against-all* pairwise similarity matrix (Figure 1B), a task that is currently carried out by the SIMAP project (29). The comparison uses Smith–Waterman alignments and compositional adjustment of the scores, as in BLAST, to prevent spurious hits between low-complexity sequence regions. Hits with bit-scores above 50 are stored and indexed in a relational database, which forms the input to the next stage of the algorithm.

### Definition of taxonomic levels

**Figure 2.**
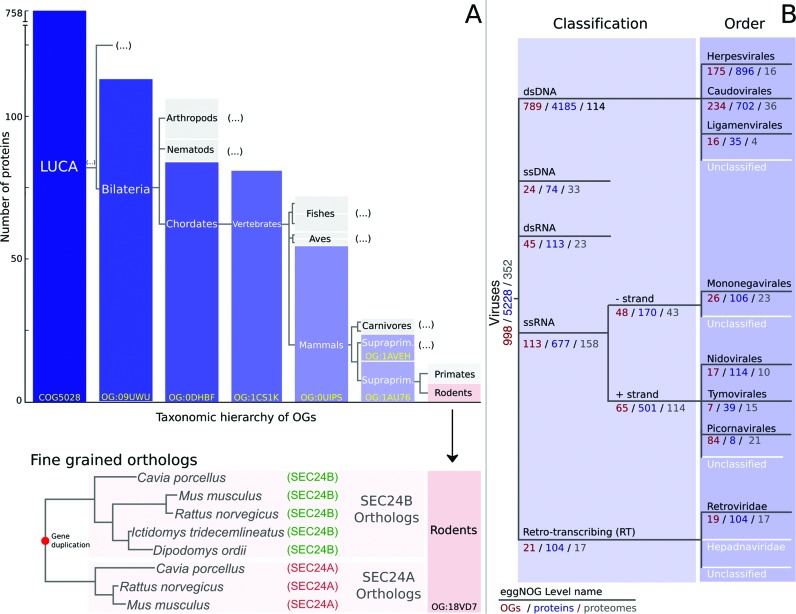
(**A**) Hierarchically consistent structure of OGs including genes from the SEC24 protein family, from the root taxonomic level (Last Universal Common Ancestor, LUCA) to the rodents specific level. Each OG is represented by a box labelled with the lineage name it belongs to, and whose size is proportional to the number of proteins grouped. Boxes filled with a blue gradient represent the nested hierarchy of OGs specifically containing the mouse SEC24A protein. Grey boxes indicate collapsed branches in the OG hierarchy. Note that another Bilateria-specific OG exist, but has been collapsed for readability reasons. The most lineage-specific OG containing the mouse SEC24A protein is at the rodents taxonomic level, which is coloured in pink. Fine grained orthology for SEC24 genes, based on the phylogenetic analysis of the 18VD7 rodent-specific group, is shown in the bottom part, with tree branches indicating a lineage specific duplication. (**B**) Viral taxonomic tree. Black branches indicate levels for which OGs were calculated, whereas white branches indicate no OG was calculated at this level. Numbers indicate the number of OGs at this level, the number of proteins contained in all OGs at this level and the number of proteomes represented by the proteins within all OGs at this level, respectively.

Because the resolution of OGs depends on the taxonomic level, the eggNOG clustering pipeline is independently executed at different predefined taxonomic levels, each spanning a different clade in the overall tree of life. Levels are manually chosen to cover evolutionarily relevant groups as well as to maximally make use of well-studied model organisms (Figure 1C). This gives rise to the hierarchical structure of the data in eggNOG (Figure 2A), where, for example, a set of mammalian sequences with a common ortholog at the base of vertebrates could be part of a single mammal-specific OG (OG:0UIPS in Figure 2A), but constitute two separate supraprimate-specific groups (OG:1AVEH and OG:1AU76 in Figure 2A). In addition, eggNOG v4.5 uses 16 predefined taxonomic levels to classify the 352 viral proteomes (Figure 2B).

### Building Orthologous Groups

eggNOG's clustering algorithm (Figure 1D) takes its basis from the manually curated Clusters of Orthologous Groups covering the three domains of life: COGs (universal with best coverage for Bacteria) (30), KOGs (Eukaryotes) (8) and arKOGs (Archaea) (31). These groups are conserved at their corresponding taxonomic level in eggNOG, and are extended with additional proteomes. For each of the predefined taxonomic levels, first, groups of in-paralogous proteins are created. Then, closely related groups of in-paralogs and single genes are merged creating clusters of homologous proteins. Such clusters can also later be split again if there is a reciprocal best hit between proteins from clusters from separate lineages. The eggNOG algorithm used to build OGs has been benchmarked and compared to similar approaches in the past using OrthoBench (6,7). Moreover, the OrthoBench test suite is regularly used to evaluate the quality of OGs every time eggNOG receives an update. Note that, although other benchmarking frameworks are available, they usually require pairwise orthology predictions, therefore preventing the correct evaluation of OGs. We have, however, incorporated such type of benchmarks to test eggNOG's new capacity to produce fine-grained predictions (see sections bellow).

### Hierarchical consistency of nested groups

The eggNOG pipeline is run independently for each of the predefined taxonomic levels considered. The imperfect quality of the proteomes used and the heuristics of the pipeline are factors that can lead to minor inconsistencies between levels, such as disagreements on when duplication events occurred for a given set of homologous proteins. This sometimes prevented the correct propagation of annotations across nested groups in previous versions, and occasionally also caused inconsistencies when applying third-party analysis pipelines to eggNOG. Version 4.5 of the eggNOG algorithm resolves this by incorporating a post-clustering step that scans all groups in all levels and eliminates inconsistencies by splitting and merging the OGs (Figure 1E). The scanning is performed in root-ward direction starting from each of the leaves in predetermined sequence. The algorithm checks for any OG that underwent the division at the parental level. If such split was found, the algorithm determines, in sequence (from largest to smallest), the species overlap between each pair of resulting groups. If no overlap is detected, the split OGs are merged together at the parental level, otherwise the proteins of the smaller group are separated from the proteins of the larger group downstream at every child level. Some apparent inconsistencies remain which reflect gene fusion events; these however represent the true mosaic history of the affected proteins and have therefore been retained. In order to examine whether the consistency pipeline had affected the quality of the groups, we have benchmarked the new, consistent, eggNOG using OrthoBench 2 (7). The benchmark results show a slight increase in the F-measure for the bilateria level (from 71.2% to 72.4%) and a small decrease for gammaproteobacteria (from 94.6% to 93.2%), indicating no major impact on the quality of groups.

### Phylogenetic analysis

Amino acid sequences from each OG at each taxonomic level are further analysed using phylogenetic methods (Figure 1G). For this release, 1.9 million phylogenetic trees were built using a slightly modified version of a previously described methodology (32,33). The currently used approach includes reconstructing multiple sequence alignments based on the consensus of several aligning and gap cleaning programs (34–38), evolutionary model testing and maximum likelihood inference (39,40). The inferred speciation events are used to derive a list of pairwise orthology predictions to make the group concept of eggNOG more comparable with pair-based orthology methods (Figure 1H); the list is provided with this eggNOG version. Finally, a Hidden Markov Model (HMM) profile is built for each group based on the untrimmed version of the multiple sequence alignment using HMMER (41) (Figure 1I), which can be used for specific OG assignments in external data sets.

### Functional annotation of orthologous groups

Once the consistency has been ensured, functional descriptions are assigned to each OG using an automated procedure (Figure 1F). At the taxonomic level of each OG, available functional annotations are collected from many sources including free-text descriptions in source genome databases, COG functional categories (8), Gene Ontology terms (42), KEGG pathways (43) and SMART/Pfam protein domains (44,45). From these, a heuristic procedure aims to identify the most descriptive shared description substring among annotated members of the group. This integrated description line is then provided together with the group as a bare-bone descriptor of what is known in terms of its role and function. While this text summary is human-readable, it cannot be used for statistical analysis, and groups are therefore also classified into the single-letter functional categories used by the COG database. The individual assignments are made by a Support Vector Machine (SVM) classifier trained on proteins within COGs, KOGs and arKOGs, using as features text description words and substrings, protein domain and Gene Ontology term assignments, as well as KEGG pathway membership information. Further technical details regarding the functional annotation pipeline are available at the methods section of the main website: http://eggnog.embl.de/#/app/methods.

## UPDATES AND ADDITIONS SINCE PREVIOUS RELEASE

During the last two years, the development work on the above computational pipeline has resulted in a series of improvements, changes and additions, aiming to make the procedure more stringent in preparation for the next major update to the underlying data set. In parallel, work on a revised web front-end and programmatic access has been undertaken, aiming to more directly address the needs of different strata of eggNOG users.

### Improved and extended functional annotations

The most common application of eggNOG remains the functional characterization of novel genes or proteins by mapping into the space of OGs for which annotations are available. Such annotations, as described above, include human-readable functional summaries as well as single-letter functional codes as defined for the COGs. With the reconciliation of nested groups at the predefined taxonomic levels, clades closer to the tips of the trees that lack annotations may now inherit from their parent groups closer to the root. In comparison to previous versions, 95 890 (5%) previously uncharacterized groups were annotated with text descriptions using this strategy, yielding 1 368 357 (72%) annotated groups in total. COG functional categories were assigned to 143 683 (7.5%) groups previously lacking them, yielding 936 917 (49%) annotated OGs in total. These cases are specifically flagged in case any application wants to exclude them. Due to the increasing need for a controlled vocabulary of functional annotations, eggNOG v4.5 provides now access to Gene Ontology, KEGG, SMART and Pfam mappings. Functional terms, as well as their relative frequencies within each group of orthologs, can be browsed interactively or queried programmatically using the API.

### Faster and more sensitive sequence annotation based on HMM models

Many applications of eggNOG build on determining which OG a novel gene falls within. Although pairwise sequence similarity tools such as BLAST (46) are extensively applied for that purpose, the use of profile Hidden Markov Models (HMMs) can provide higher sensitivity for detecting remote similarities and overall performs better in large data sets (41). Moreover, the structure of eggNOG is particularly suitable for HMM analysis, as the hierarchical taxonomic structure of nested OGs allows adjusting the search to use the most appropriate level for each analysis. Users can choose to increase the resolution of mappings and annotations by restricting searches to lineage-specific levels, thus maximizing sequence similarity within groups, and therefore have access to better-quality multiple sequence alignments and HMMs. In eggNOG v4.5, 1.9 million HMMs have been reconstructed based on the complete set of OGs at all taxonomic levels. A collection of raw HMM files is available for download for each level to annotate external data sets. Furthermore, three optimized databases are provided that cover the three domains of life: Archaea, Bacteria and Eukaryota. These three databases have been designed to contain a selection of HMMs where larger OGs at the deepest taxonomic levels have been split into their corresponding lineage-specific, but more fine-grained, OGs.

### Annotation of viral orthologous groups

Viruses have not been taxonomically or functionally annotated in other orthology resources. In this version of eggNOG, we cover non-cellular life for the first time, by the addition of viral orthologous groups constructed analogously but in parallel to the rest of the genomes covered by eggNOG. The viral proteins were processed by the standard eggNOG pipeline, which was seeded with phage orthology groups published in Kristensen et al. (47), analogous to how COGs, KOGs and arKOGs are used to seed each cellular domain. An additional step to merge orthology groups was performed on those viral OGs where a majority of the included proteins had the same Pfam domain architectures at the clan level. This resulted in 2605 final viral orthology groups at the top level covering 5228 proteins from 352 proteomes. Viral OGs have been calculated at 16 separate levels within the virus taxonomy (Figure 2B). Viral OGs, along with their associated HMMs, multiple sequence alignments, phylogenetic trees and functional annotations are available for downloading.

### Pairwise orthology predictions

Although eggNOG currently has more than a hundred predefined taxonomic levels of orthology resolution, it is not infrequent that OGs contain in-paralogs and masked co-orthology relationships at some levels, the more the closer to the root of the tree of life, particularly at the deepest taxonomic levels. This is irrelevant when the intended analysis focuses on using the functional description of OGs, such as for the annotation of genomic and metagenomic data (48–50). However, accurate distinction among one-to-one, one-to-many and many-to-many relationships is often needed to address evolutionary questions such as the reconstruction of the tree of life (51), estimating the relative age of sequences (52), or studying gene duplication (53). For this reason, eggNOG v4.5 allows refining the content of each OG through the automated analysis of precomputed phylogenetic trees. This allows us to extract fine-grained orthology relationships among the protein members of each OG, even when the maximum level of taxonomic resolution is reached in the OGs hierarchy (Figure 2A). The performance of pairwise orthology predictions from eggNOG was recently evaluated as part of the Quest for Orthologs Benchmarking initiative, showing comparable results to other pairwise orthology resources (http://orthology.benchmarkservice.org).

### New web interface: faster searches and advanced data browsing

**Figure 3.**
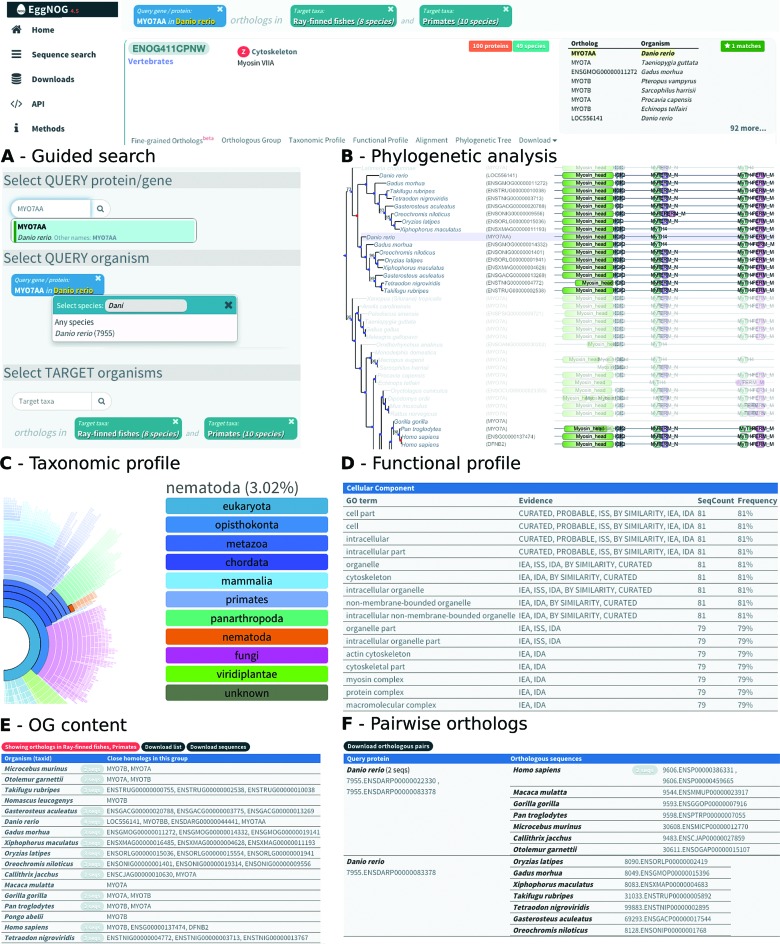
Website screenshots showing fish and primate orthologs for the myosin protein MYO7AA. (**A**) The guided search dialog used to retrieve the orthologs. (**B**) Partial tree representation of the associated phylogenetic tree. Blue nodes in the tree represent speciation events. Red nodes indicate duplication events (in-paralogs). Pfam domains are shown in-line for all the orthologous sequences. Note that tree visualization is adapted to the query, highlighting the seed and target species and graying out the rest. (**C**) Taxonomic profile representation showing the distribution of orthologs in the tree of life. (**D**) Functional profile based on Gene Ontology terms associated to the OG. (**E**) Filtered content of the OG (protein names and sequences), restricted to the query and target species. (**F**) Pairwise orthology predictions adapted to the query protein and the target species. In-paralogy and co-orthology relationships are resolved according to the speciation and duplication events inferred from the phylogenetic tree.

All the described improvements have been fully integrated into the new eggNOG back-end database and the completely redesigned front-end web interface (Figure 3), which enables much faster searches and provides many new browsing and visualization capabilities. Special efforts have been made to facilitate the access to eggNOG data for less experienced users. The default search panel (Figure 3A) allows for guided queries in three simple steps. First, users enter a protein or gene name, which is instantly searched and autocompleted based on an in-house ID translation database covering all major sequence providers. Second, users are asked to disambiguate the source organism for the selected protein, which allows distinguishing between genes having the same name in different species (i.e. CDK1 in human versus CDK1 in chimp) and enables eggNOG to infer pairwise orthology mappings. Finally, users can provide a list of target organisms, or complete lineages, from which they would like to retrieve orthologs. This ensures an interpretable output and allows eggNOG to automatically select the most appropriate taxonomic level for the given query. For example, if a query is set to find rat orthologs of mouse CDK1, eggNOG will automatically retrieve the corresponding OG at the *rodents* taxonomic level and limit the displayed results to rat and mouse proteins only.

Finally, five new information channels have been added to the interface, which permit users to browse the extended data associated with each OG: (i) *Phylogenetic trees* and *Alignments* provide an integrative overview of the evolutionary relationships of all member proteins within each OG together with their functional annotations. The phylogenetic tree image (Figure 3B) highlights the query and target sequences, aligned domain regions, and the inferred duplication and speciation events within the group. (ii) The *Taxonomic Profile* channel (Figure 3C) offers a visual overview in the form of a *sunburst* representation about the distribution of orthologs across different taxonomic subdivisions. (iii) The *Functional Profiles* allow to inspect the frequency of functional terms, domains and pathways found within each group (Figure 3D). (iv) The *Orthologous Group* channel (Figure 3E) displays the complete list of members in a group, filtering out any species that are not present in the query and allowing users to download both ortholog names and sequences in FASTA format. (v) The *Pairwise Orthology* provides a refined list of orthologs to the specific queried protein, including fine-grained delineation of one-to-one, one-to-many and many-to-many relationships (Figure 3F).

### Programmatic access

A scalable RESTful web service has been implemented that permits programmatic access to all eggNOG data, as well as their integration in third party resources. It currently supports queries to retrieve complete OGs, protein sequences, alignments, phylogenetic trees, HMM models and functional profiles in text and JSON formats. When a particular protein name is fixed as a query, pairwise orthology predictions can also be fetched using the web service API.

## CONCLUSIONS

With the changes, updates and additions described above, eggNOG v4.5 provides one of the most complete and scalable databases for orthology prediction and functional annotation publicly available. The introduction of hierarchical consistency between groups, and the ability to stringently derive pairwise orthology relationships, brings the possibility of using eggNOG data both for large-scale sequence annotation projects and for evolutionary analyses requiring finer resolution. The redesign of the website frontend and backend databases offers fast and seamless integration with all eggNOG data, which ultimately aims at covering a variety of use-cases and users. Finally, the extensive changes described here enable more efficient and regular incorporation of newly sequenced high quality genomes to keep comprehensive species coverage.
